# Supplementary figures and images for: Structural insight into the role of novel SARS-CoV-2 E protein: A potential target for vaccine development and other therapeutic strategies
Source: PLoS One. 2020 Aug 12;15(8):e0237300. doi: 10.1371/journal.pone.0237300 (PMC7423102; doi:10.1371/journal.pone.0237300)

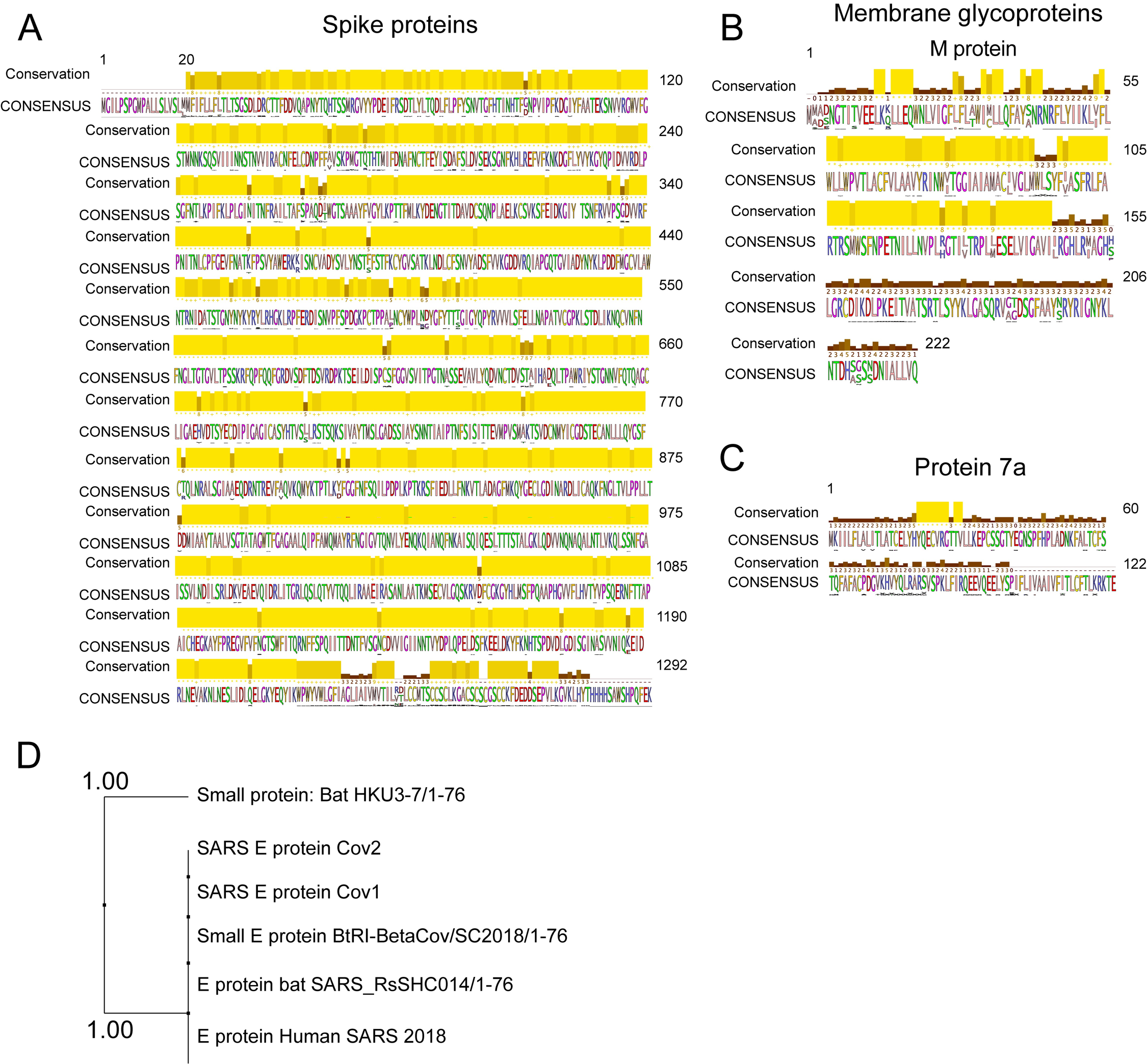

Supplement: S1 Fig — (A) Conservation score and consensus sequence obtained from multiple sequence alignment for spike protein after a BLASTp search. (B) Conservation score and consensus sequence obtained from multiple sequence alignment for M-protein after a BLASTp search. (C) Conservation score and consensus sequence obtained from multiple sequence alignment for protein 7a after a BLASTp search. (D) Clustering showing close association of the sequences aligned in panel B and their sequence distance. (TIF) [file pone.0237300.s001.tif]

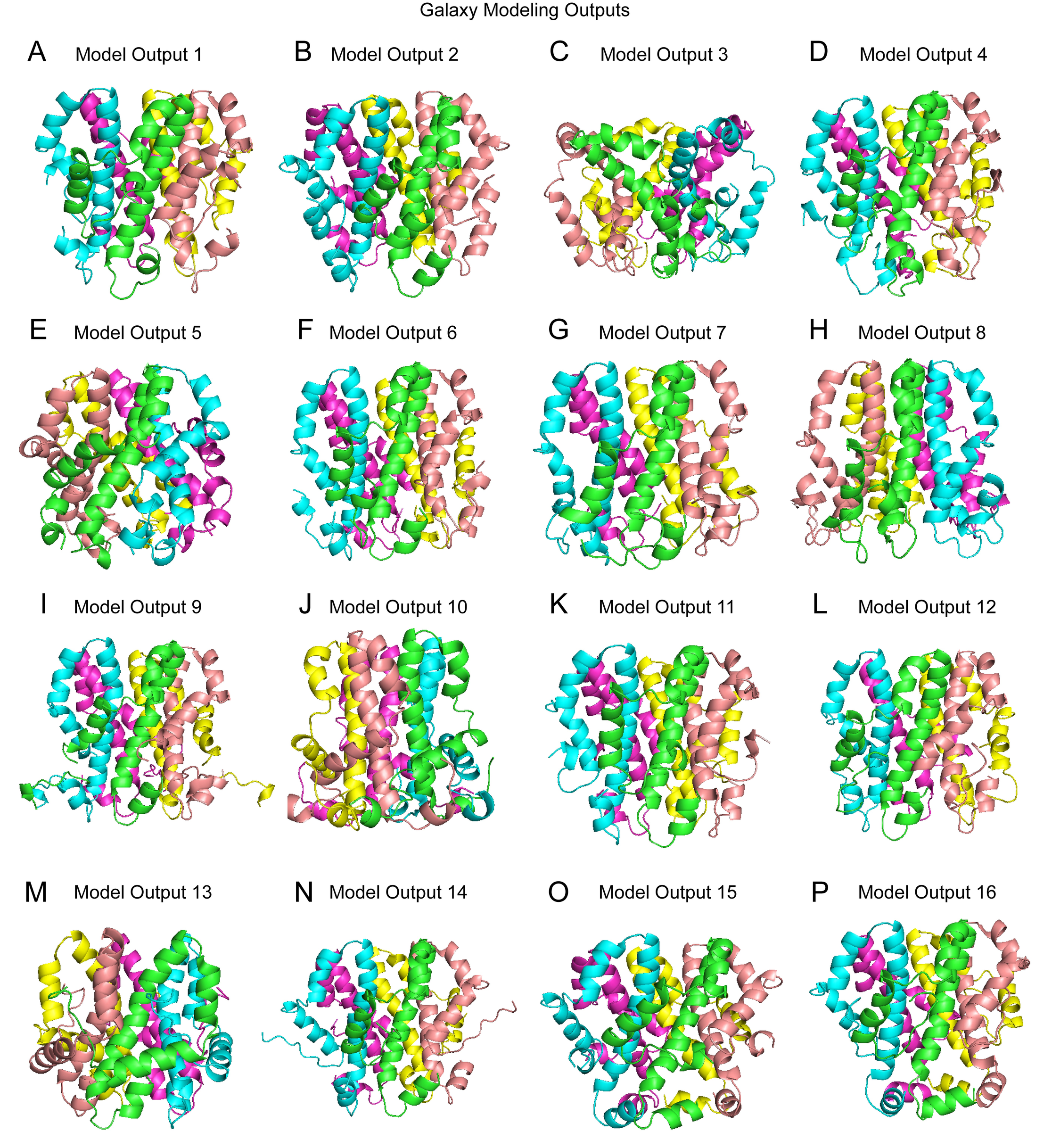

Supplement: S2 Fig — (TIF) [file pone.0237300.s002.tif]

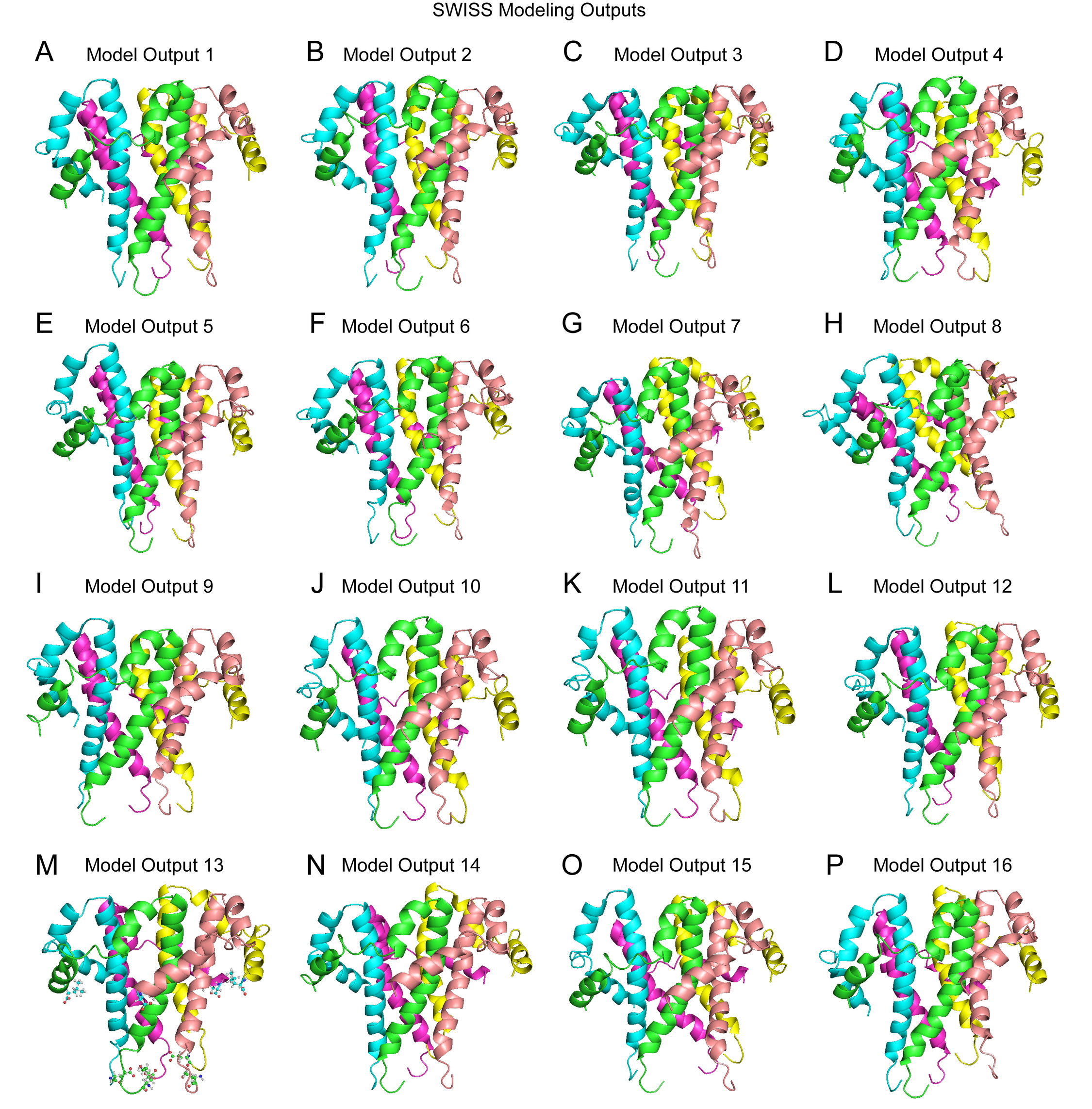

Supplement: S3 Fig — (TIF) [file pone.0237300.s003.tif]

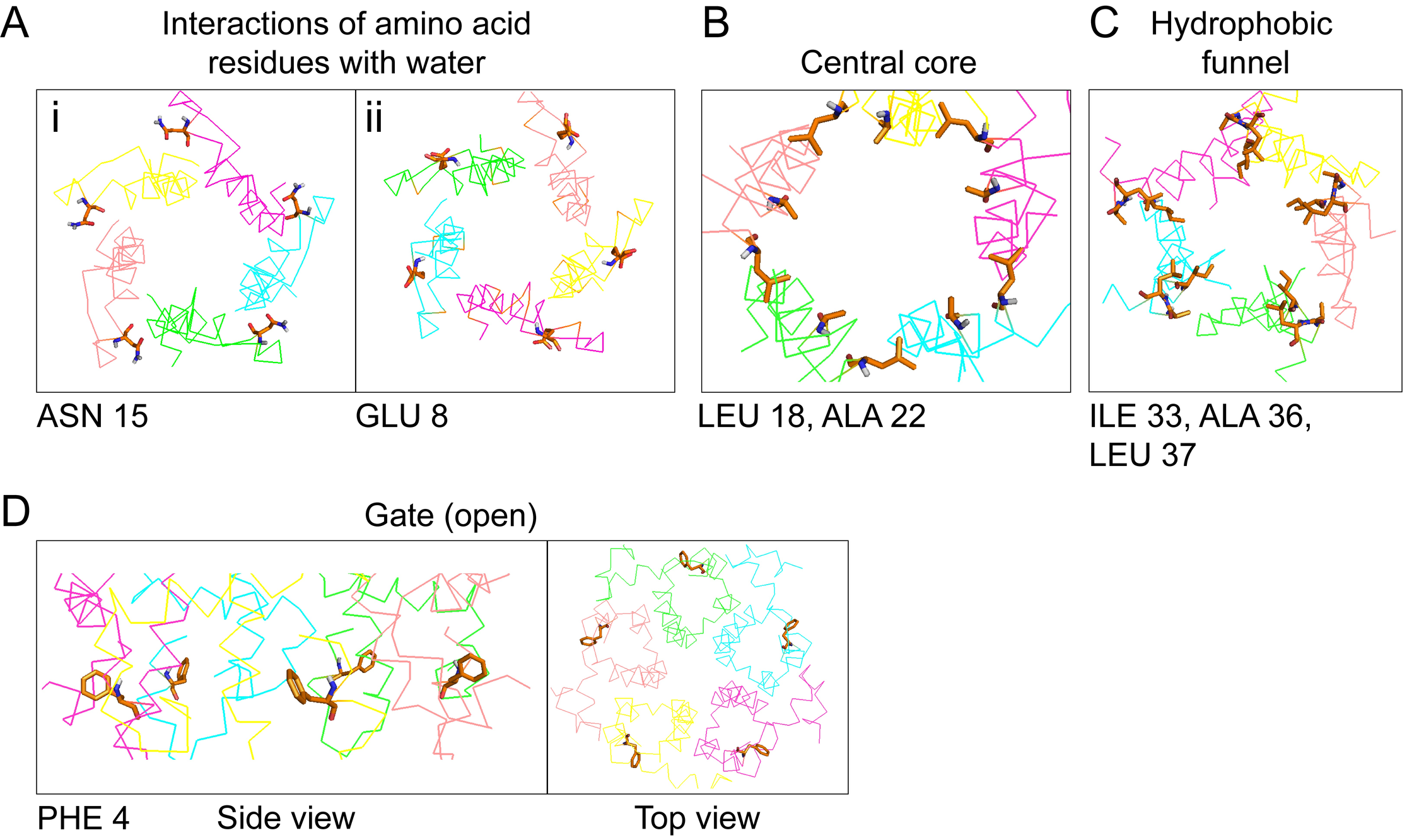

Supplement: S4 Fig — (A) Top view of residues’ orientation of the E-put protein generated from PYMOL (i) ASN 15 (ii) GLU 8. (B) Top view of residues’ orientation of the central core of the E-put protein generated from PYMOL. (C) Top view of residues’ orientation of the hydrophobic funnel of the E-put protein generated from PYMOL. (D) Residues’ orientation generated from PYMOL (i) side view (ii) top view showing the Gate of the E-put protein in the open conformation. (TIF) [file pone.0237300.s004.tif]

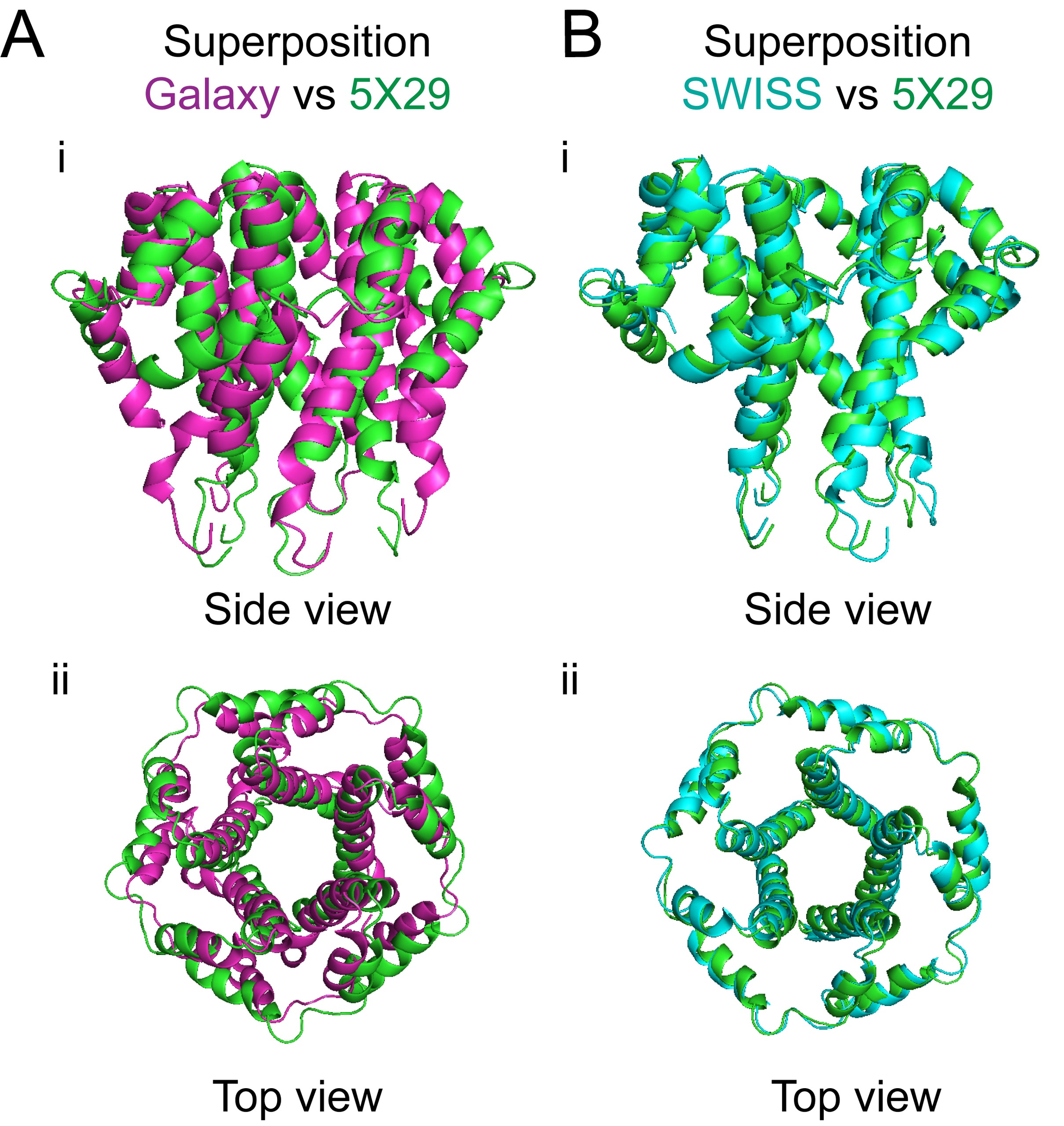

Supplement: S5 Fig — (A) Superposition of the E-put and the 5X29 CoV-1 template protein (purple: E-put; green: 5X29) as (i) side view and (ii) top view. (B) Superposition of the SWISS-MODEL and the 5X29 CoV-1 template protein (green: 5X29; cyan: SWISS-MODEL) as (i) side view and (ii) top view. (TIF) [file pone.0237300.s005.tif]

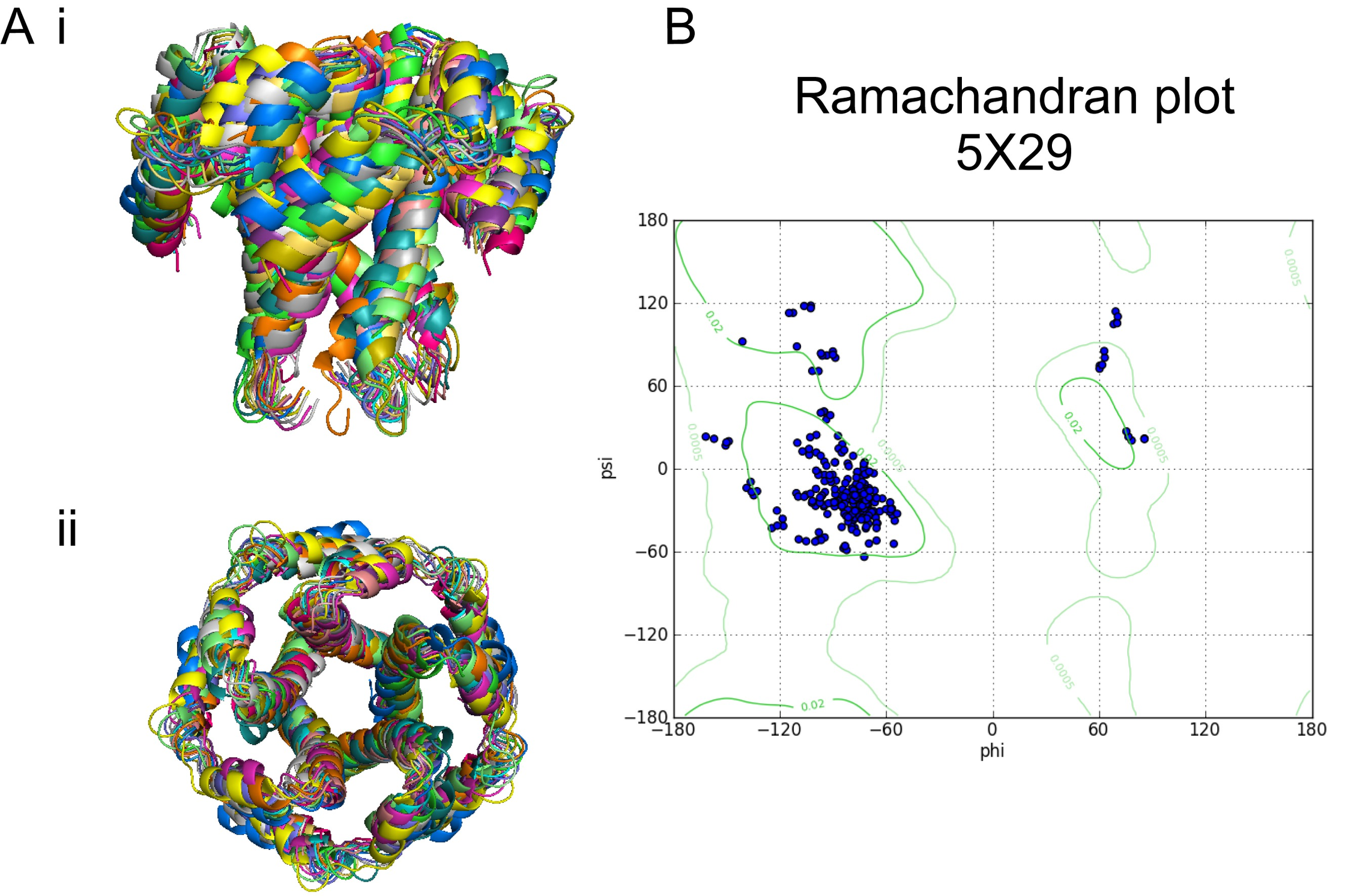

Supplement: S6 Fig — (A) Structure ensembles of the 5X29 NMR structures as (i) side view and (ii) top view. (B) Ramachandran plot of the 5X29 NMR structure of the CoV-1protein (template). (TIF) [file pone.0237300.s006.tif]

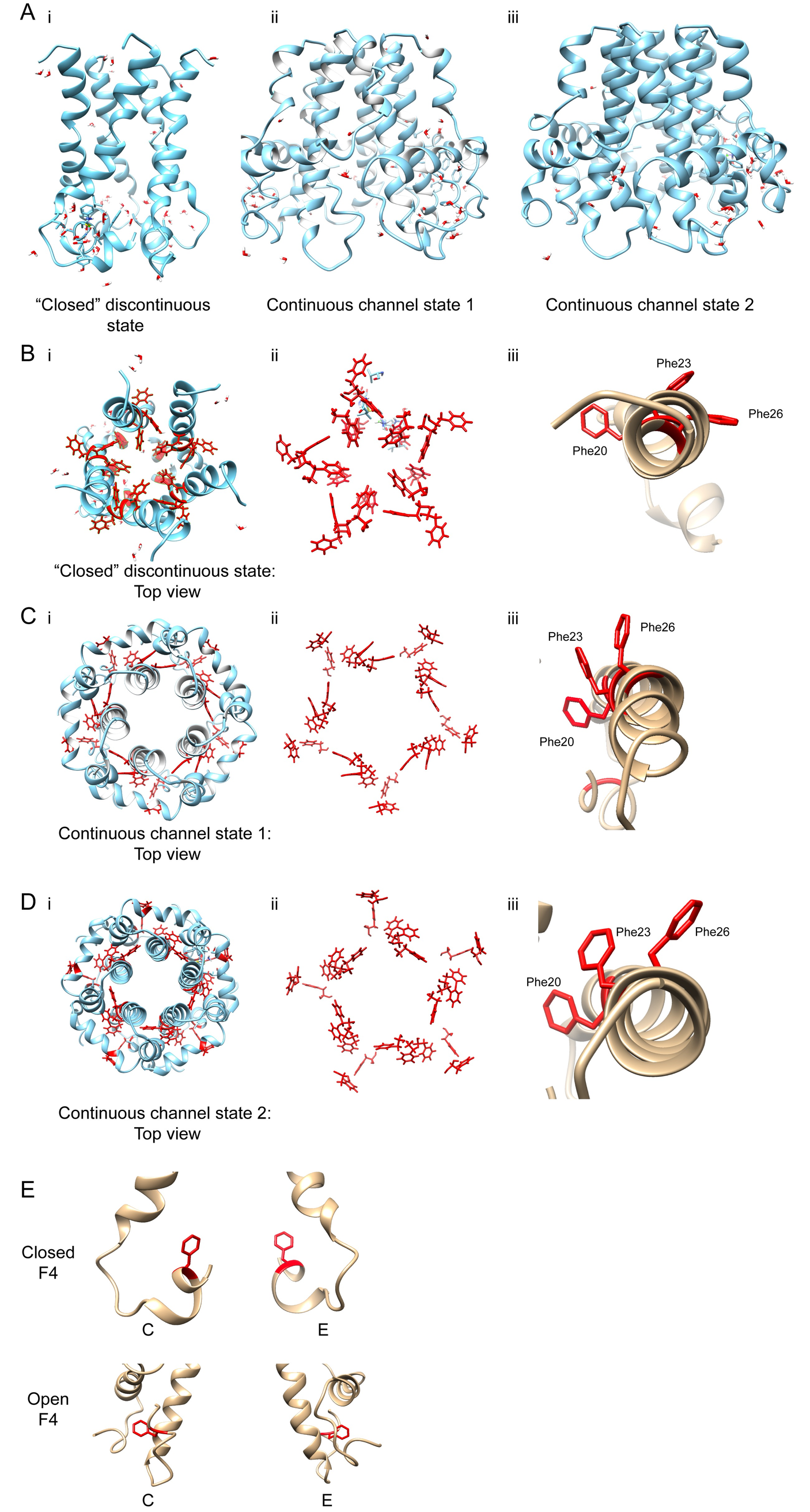

Supplement: S7 Fig — (A) Docking of water to the modeled structure of E-put protein in its different conformations (i) Closed discontinuous state (ii) Continuous channel state 1 (iii) Continuous channel state 2. (B) (i-ii) Top view showing all five bottleneck phenylalanine residues (PHE 26) along with adjacent phenylalanine residues (PHE 23 and PHE 20) for the closed discontinuous state. (iii) Enlarged image of the top view orientation of the PHE 26, PHE 23, and PHE 20 on the E chain. (C) (i-ii) Top view showing all five bottleneck phenylalanine residues (PHE 26) along with adjacent phenylalanine residues (PHE 23 and PHE 20) for the open continuous channel state 1. (iii) Enlarged image of the top view orientation of the PHE 26, PHE 23, and PHE 20 on the E chain. (D) (i-ii) Top view showing all five bottleneck phenylalanine residues (PHE 26) along with adjacent phenylalanine residues (PHE 23 and PHE 20) for the continuous channel state 2. (iii) Enlarged image of the top view orientation of the PHE 26, PHE 23, and PHE 20 on the E chain. (E) The orientation of the PHE 4 residues of the Gate of the E protein generated in CHIMERA in its closed and open conformational state. (TIF) [file pone.0237300.s007.tif]
